# Using AI in healthcare education: a rapid review and commentary

**DOI:** 10.3389/fresc.2026.1716654

**Published:** 2026-03-09

**Authors:** Razan Hamed, Dylan Van

**Affiliations:** Department of Rehabilitation and Regenerative Medicine, Vagelos College of Physicians and Surgeons, Columbia University, New York City, NY, United States

**Keywords:** healthcare, rehabilitation, competency based education, artificial intellegence technology, critical thinking

## Abstract

Artificial intelligence (AI) is rapidly transforming healthcare and rehabilitation education, offering new pathways for personalized learning, adaptive assessment, and simulation-based training. This paper provides a rapid review and commentary on current research exploring AI’s integration into healthcare curricula, highlighting its potential to enhance competency development, critical thinking, and learner engagement. Evidence shows that AI can enrich educational experiences by tailoring instruction to individual needs, facilitating clinical reasoning, and reducing the cognitive and logistical burdens faced by graduate students who balance academics with professional and personal responsibilities. Yet, the increasing reliance on AI also introduces ethical, cultural, and pedagogical challenges, including algorithmic bias, data privacy concerns, inequitable access to technology, and the risk of diminishing independent judgment. Within rehabilitation education, additional issues arise related to patient confidentiality, assessment authenticity, and the unauthorized use of educators’ intellectual property. The findings emphasize that successful integration of AI in healthcare education depends on proactive strategies that uphold ethical practice, equity, and reflective learning. By embedding AI literacy, cultural humility, and clear ethical guardrails into curricula, educators can ensure that technology complements—rather than compromises—the humanistic and critical dimensions of healthcare practice.

## 1 Introduction

Healthcare educators strive to prepare competent professionals who can meet the evolving needs of their communities and deliver high-quality care (1). Graduate programs in medicine, nursing, and allied health professions achieve this mission through competency-based education, inclusive teaching practices, and integration of innovative instructional technologies (2–6). Multifaceted efforts are put in place to rail guard the teaching and learning process toward competency. For example, accreditation standards at national, local, and regional levels guide these efforts, shaping curricula, learning activities, and assessments to foster independent learners, creative thinkers, and critical appraisers of evidence-informed practice (7–9). Other measures related to competence include board certification and licensure exams, continuing education modules, professional development requirements, capacity building and professional trainings in healthcare aim to preparing competency healthcare practitioners (10–13).

However, regardless of the measures that assess the effectiveness of teaching and instruction toward competency, a key tenet to competence is innate the student's capacity for critical thinking and independent learning. Educator should not only consider the finess of their curricula and instructional design but also other factors affecting how students are learning and work toward competency.

**Figure 1:**
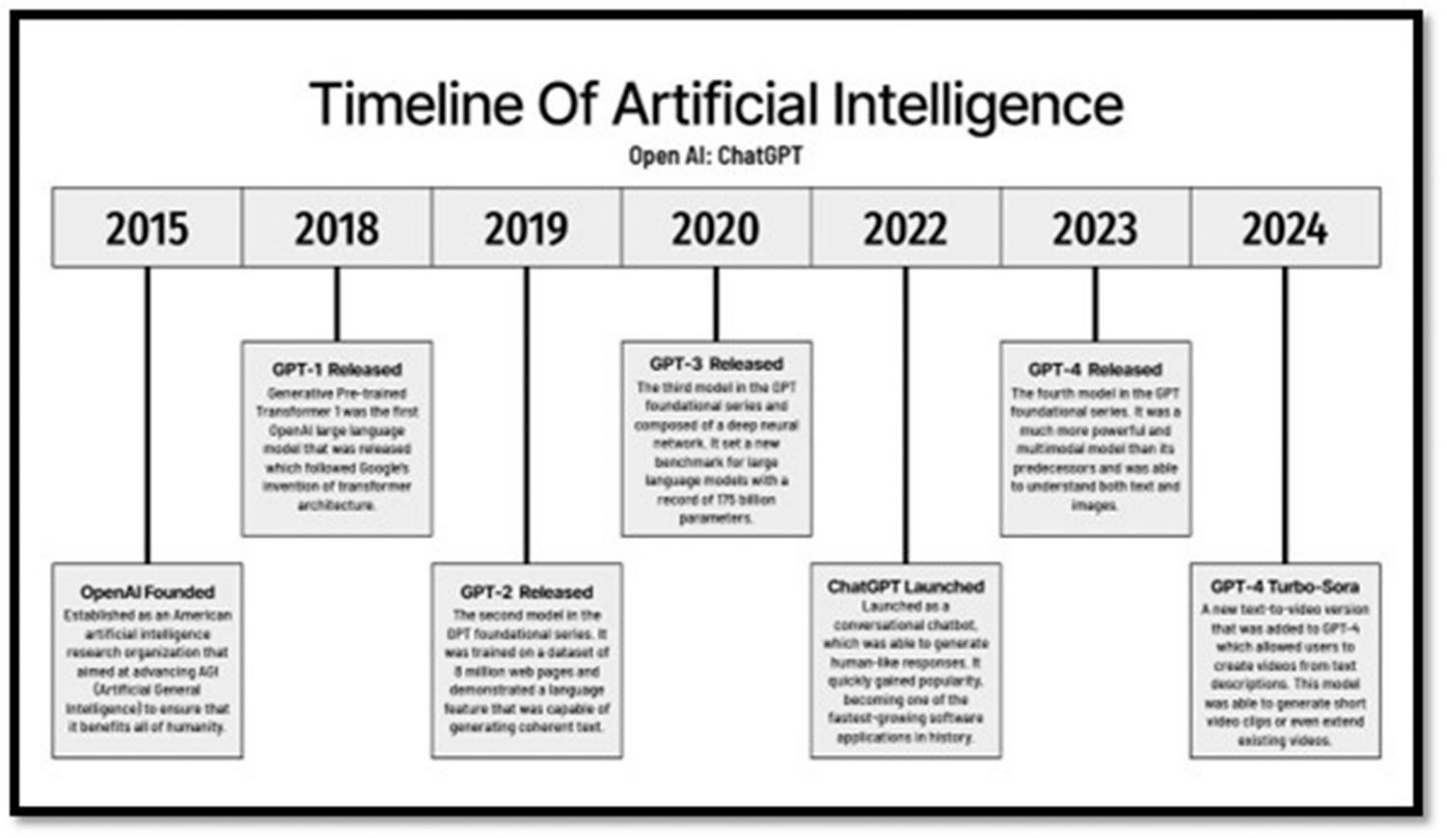
Timeline for open AI development (14).

Artificial intelligence (AI) has rapidly emerged as both a tool and a challenge for educators especially in healthcare. The timeline of artificial intelligence presented in the figure illustrates the rapid evolution of OpenAI's generative language models and provides essential context for understanding the integration of AI in healthcare education (Figure 1). Beginning with OpenAI's founding in 2015, the organization aimed to advance artificial general intelligence for the benefit of humanity. Over the following decade, successive model releases—from GPT-1 in 2018 through GPT-4 Turbo-Sora in 2024—demonstrated exponential growth in computational power, multimodal capability, and interactivity (14). Each iteration, particularly GPT-3 and GPT-4, represented a leap toward more human-like understanding and communication, enabling applications far beyond text generation. Within healthcare education, these advancements underpin today's AI-driven simulations, virtual tutors, and adaptive feedback systems that support learners in complex domains such as anatomy, neuroscience, and rehabilitation sciences. As discussed in this paper, the same technologies driving conversational AI—natural language processing, multimodal reasoning, and intelligent feedback loops—now shape how students learn, practice, and reflect in digital environments. Understanding this timeline underscores that AI's educational impact is not accidental but the product of years of iterative innovation that continues to redefine competency development, access, and ethics in healthcare and rehabilitation training.

While AI can offer a myriad of benefits to both educators and learners (e.g., streamline assignments, exams, and projects its influence raises pressing concerns and questions. For example, how does AI impact students' development of critical thinking? What are the long-term implications for clinical care when healthcare education increasingly incorporates AI?

This paper provides a rapid review exploring the current research on the integration of AI in healthcare education. We highlight its potential benefits and risks, summarize key studies, and offer practical recommendations for educators seeking to balance innovation with the preservation of essential human competencies.

## 2 Opportunities and risks of AI in healthcare education

### 2.1 Competency and critical thinking

Competency-based education in health professions requires explicit domains addressing emerging technologies. Garvey et al. (38) noted that few studies assess clinician knowledge, skills, or attitudes regarding AI, revealing significant gaps in competency frameworks. They urge co-development of pragmatic, testable competencies by educators and health systems to better prepare students for AI-enabled practice. Kleib et al. (15) further argue that digital health education remains fragmented, with inconsistent approaches to emerging technologies like AI, robotics, and virtual reality. They recommend transitioning curricula from eHealth to a broader digital health focus, ensuring alignment with contemporary clinical realities.

AI applications are poised to transform healthcare education by enabling personalized learning pathways. For example, AI can tailor instructional content to students' level of competency by providing tools toward expected competencies (e.g., practice quizzes, synthesis or research, summary and breakdown of complex readings (16).

Additionally, AI can offer immersive learning experiences that offer convenient learning environment for students such as simulated virtual patients, intelligent tutoring systems, and adaptive testing environments. These tools promote improvements in learner engagement, critical thinking, and clinical decision-making (17, 18). By offering real-time feedback and contextualized clinical scenarios, AI helps bridge the gap between theoretical knowledge and clinical application.

Importantly, AI's influence on critical thinking is twofold. On one hand, AI facilitates evidence-informed inquiry and expands learners' ability to synthesize information efficiently. On the other, uncritical reliance on AI-generated outputs can undermine independent reasoning, creativity, and reflective judgment. As Mir et al. (19) emphasize, AI should serve as a cognitive partner that augments—not replaces—human judgment. Developing ethical guardrails, such as critical appraisal training and AI literacy competencies, is essential to ensure that AI integration enhances rather than diminishes learners' problem-solving and decision-making capacities (20).

Despite its promise, responsible integration of AI requires addressing ethical, practical, and pedagogical challenges. Concerns about algorithmic bias, data privacy, cost of implementation, and the limited preparedness of faculty to design and oversee AI-enabled instruction remain substantial barriers (21, 22). Educators must therefore balance innovation with safeguards that ensure equity, transparency, and accountability in the use of AI-driven tools.

### 2.2 Student perspectives and competencies

From the student perspective, artificial intelligence (AI) is emerging as both a source of excitement and uncertainty in healthcare education. Research shows that graduate healthcare students generally hold positive attitudes toward AI but report limited knowledge and practical skills (23, 24). For many students in rehabilitation and allied health programs, attaining a professional degree already involves juggling demanding coursework, long clinical hours, financial pressures, and family obligations—all while adapting to evolving healthcare technologies and sociopolitical expectations (25, 26). As a result, AI offers the promise of flexibility—a tool that can personalize learning, simulate patient scenarios, and provide feedback on complex clinical reasoning tasks when instructors or peers are unavailable. Students often describe these tools as empowering because they allow them to learn “anytime and anywhere,” easing the tension between academic expectations and real-life responsibilities (26).

From this vantage point, students see AI as a way to lighten the load of navigating healthcare education. Adaptive learning platforms and intelligent simulations can save time by offering instant feedback and individualized learning trajectories, helping students focus on mastery instead of catching up on missed material (17, 27). For example, in a study by Kim et al. (28), rehabilitation science students using AI-driven virtual patients reported greater confidence in assessment and treatment planning compared to those using traditional materials. Similarly, medical students using AI-supported anatomy and physiology software achieved equivalent outcomes to lab-based learning but at a lower cost and with greater schedule flexibility (29). From the learner's standpoint, these benefits can translate into reduced burnout and enhanced autonomy, especially for those balancing multiple personal and academic demands.

However, access to AI tools is not equally distributed among students. Access to reliable technology, internet connectivity, and costly AI subscriptions can limit who truly benefits from these innovations (22). Some learners, particularly those from lower socioeconomic or under-resourced backgrounds, describe frustration and exclusion when AI-based learning tools are introduced without institutional support. For example, in Al-Qaysi et al.'s (2024) survey, nursing students in low- and middle-income regions expressed concern that AI adoption might favor those with better digital literacy or access to newer devices. From a student's perspective, these disparities underscore the importance of educators and institutions recognizing digital inequity as an ethical issue in AI integration. Students consistently call for equal access to technology, transparent use of AI in evaluation, and training that empowers—not alienates—them in this transition to AI-driven learning.

Taken together, these findings reflect a student-centered call for AI literacy and inclusion in healthcare curricula. Students want to understand how AI works, how to use it ethically, and how to retain human judgment in an increasingly automated environment. As De Gagne et al. (30) and Teng et al. (31) note, this includes integrating topics such as digital professionalism, cyberethics, and prompt design into the classroom. From the student point of view, AI is not simply a technological advancement—it is a mirror of their lived experience in a demanding educational environment where adaptability, empathy, and access matter as much as intelligence itself.

### 2.3 Ethical and cultural considerations

From a student's rehabilitation perspective, the rise of AI brings both opportunity and ethical complexity. While AI systems can provide adaptive learning, simulate patient interactions, and enhance clinical reasoning practice, their integration into healthcare education also introduces ethical dilemmas that are especially salient in rehabilitation—where empathy, client-centeredness, and professional judgment are essential competencies (32).

A major ethical concern involves assessment integrity. AI-assisted platforms can generate responses, treatment plans, and reflective writing that mimic authentic student output. This raises challenges in evaluating students' true competence and clinical reasoning skills (30, 33). In rehabilitation education, where critical thinking and creativity underpin occupational and physical therapy practice, overreliance on AI tools during assignments may blur the boundary between assistance and academic dishonesty. Educators are therefore called to establish transparent AI-use policies, design authentic assessments that measure human reasoning, and include training on academic integrity in the AI era (34).

Another ethical issue centers on data privacy and patient confidentiality. Many AI-driven learning systems rely on large datasets—including de-identified patient information or simulated cases derived from real-world data—to teach diagnostic and therapeutic reasoning. However, inadequate data governance can expose sensitive rehabilitation data, such as patient progress reports, functional scores, or video-based movement analysis (39). Students using AI tools that process or generate patient scenarios must understand the ethical and legal boundaries of data sharing, particularly under frameworks like Health Insurance Privacy and Accountability Act (HIPAA) and GDPR (40). Training in data stewardship and confidentiality is therefore not optional—it is integral to professional formation in rehabilitation disciplines.

A third area of concern involves the use of professors' intellectual property. Some AI tools are trained on digital learning materials—such as lecture slides, textbooks, and recorded demonstrations—without consent from educators. This raises questions of authorship, ownership, and academic fairness. Rehabilitation educators often produce proprietary teaching content grounded in clinical expertise, including intervention frameworks, patient videos, or adaptive equipment design models. When such materials are scraped or replicated by generative AI models, students may unknowingly engage with unlicensed or misattributed content. Ethical AI integration in rehabilitation education thus requires institutional policies that protect intellectual property, ensure consent for data use, and teach students about respecting creators’ rights (22).

Finally, there are broader concerns about bias and representation. If AI models are trained predominantly on Western, able-bodied, or monocultural rehabilitation data, they may reinforce clinical stereotypes or overlook the lived experiences of patients from diverse cultural or disability backgrounds (35). For students, this can distort their understanding of inclusive, occupation-centered care. Embedding discussions of AI bias and cultural humility in coursework helps prepare rehabilitation students to critically evaluate AI-generated outputs and advocate for equitable technology use in clinical practice.

In short, while AI can enhance learning efficiency and clinical preparedness, its ethical integration into rehabilitation education must prioritize academic honesty, data privacy, intellectual property protection, and cultural equity. Students, educators, and institutions share responsibility for cultivating digital professionalism and moral reasoning alongside technical competence.

## 3 Responsible integration of AI in rehabilitation education: practical recommendations for educators

At this stage in the evolution of artificial intelligence, its integration into healthcare education is no longer optional but inevitable. Educators in rehabilitation sciences must embrace and proactively adopt ethically grounded AI strategies into their curricula. Doing so not only enhances student engagement and learning efficiency but also ensures that technological innovation aligns with the values of patient-centered care and professional accountability.

### 3.1 Collaborative projects

Collaboration between rehabilitation educators and AI engineers offers a powerful avenue to design AI-enabled coursework and experiential projects. For example, faculty and computer science partners might co-develop a machine-learning tool that analyzes patient movement data to detect gait abnormalities—allowing students to explore clinical decision-making using real-world datasets (32). Interdisciplinary capstone projects can also engage occupational therapy students in designing adaptive AI systems that personalize therapy plans for clients with stroke or traumatic brain injury. Such collaborations cultivate students' digital literacy and deepen their appreciation for innovation grounded in clinical ethics.

### 3.2 AI literacy

Building AI literacy among students and faculty is essential to responsible integration. Educators can provide accessible resources—such as video tutorials, short modules, or peer-led workshops—that explain how AI algorithms function and what their limitations are (22). In rehabilitation contexts, this may involve demonstrations of how predictive analytics can inform therapy intensity or outcome forecasting for musculoskeletal rehabilitation. When students understand the “why” behind AI outputs, they can more critically and confidently apply these tools in evidence-based practice.

### 3.3 Critical appraisal

Rehabilitation students should be trained to critically evaluate AI-generated outputs against human-verified evidence. For example, when an AI system recommends an exercise progression for a patient with Parkinson's disease, students can compare those suggestions to established practice guidelines from the American Physical Therapy Association (APTA). This activity helps learners recognize algorithmic bias and error while reinforcing their accountability for clinical reasoning (33). Embedding such exercises in assignments transforms AI from a passive tool into a stimulus for higher-order thinking and professional discernment.

### 3.4 Metacognition and reflection

AI can also serve as a reflective mirror for students' own reasoning. Integrating AI-generated case analyses into classroom discussions allows students to compare their decision-making with algorithmic outputs, prompting metacognitive reflection on strengths, biases, and ethical choices (30). For instance, after using an AI tool to create a rehabilitation plan for a patient with spinal cord injury, students can reflect on how empathy, cultural context, or client motivation—factors AI might overlook—affect therapeutic outcomes. Such reflective comparisons foster humility, self-awareness, and ethical sensitivity.

### 3.5 Ethical guardrails

To prevent misuse, educators must establish clear ethical guardrails for AI use in coursework, simulations, and clinical reasoning assessments. Policies should outline acceptable levels of AI assistance, proper citation of AI-generated material, and patient privacy standards when using data-driven platforms (36). In rehabilitation programs, this might mean restricting AI use for patient documentation unless the data are anonymized, or mandating disclosure when AI tools assist in clinical report generation. Aligning these rules with professional codes of ethics ensures that technological fluency never supersedes ethical practice.

### 3.6 Cultural humility

AI systems often reflect the cultural and demographic biases of their training data, which can skew clinical recommendations. Educators should embed cultural sensitivity and digital equity into AI instruction by encouraging students to examine how these systems interpret patient diversity. For example, a rehabilitation AI tool might misclassify movement patterns in patients from different ethnic groups due to underrepresentation in its datasets (35). Classroom discussions and role-play scenarios can help students recognize these limitations and advocate for inclusive, bias-aware AI tools that respect patient rights and dignity.

### 3.7 Shift in focus

Finally, the integration of AI should allow educators and students to refocus on human connection and deep learning. By automating administrative or repetitive learning tasks—such as grading quizzes or tracking patient metrics—AI can free time for mentorship, clinical reasoning development, and wellness activities (27). For rehabilitation students, this shift allows more focus on empathy, creativity, and problem-solving with real clients, reinforcing the humanistic core of the profession.

## 4 Pros and cons of using AI in graduate education

The integration of AI in graduate healthcare education brings both opportunities and challenges. Table 1 provides a concise overview of the key advantages and challenges of integrating artificial intelligence (AI) in graduate healthcare and rehabilitation education. On the positive side, AI supports personalized and adaptive learning pathways, allowing students to learn at their own pace while receiving targeted feedback on clinical reasoning and performance. These tools also enhance access to evidence-based information, enabling rapid retrieval of current research to inform decision-making. Furthermore, simulation-based training powered by AI creates safe, realistic opportunities for students to practice complex rehabilitation procedures before working with actual patients. Automation of routine or repetitive tasks—such as grading quizzes, tracking clinical competencies, or managing patient documentation—frees both students and faculty to focus on higher-order learning, mentorship, and reflection. Collectively, these advantages demonstrate AI's potential to modernize curricula, enhance efficiency, and embed AI literacy into the professional preparation of future clinicians (17, 27).

**Table 1:** Key pros and cons of using AI in graduate education.

Pros	Cons
Personalized and adaptive learning pathways	Risk of overreliance, reducing independent judgment
Enhanced access to evidence and rapid information retrieval	Potential erosion of critical thinking and creativity
Simulation and training opportunities that augment clinical readiness	Ethical concerns including bias and transparency
Reduction of rote or repetitive tasks	Faculty preparedness and training gaps
Support for innovative teaching approaches	High costs of implementation and maintenance
Opportunities to integrate AI literacy into curricula	Student anxiety about job security

However, the table also highlights important cautions. Overreliance on AI tools risks diminishing independent judgment and creative problem-solving—skills central to rehabilitation practice. Students and faculty alike may face ethical challenges, including bias in algorithms, lack of transparency in AI decision-making, and unequal access to digital resources (22, 33). Moreover, limited faculty training, high implementation costs, and uncertainty about job security in an AI-augmented workforce can generate resistance and anxiety among learners. These concerns underscore the need for balanced adoption strategies that pair innovation with accountability.

Ultimately, the takeaways from Table 1 suggest that the promise of AI in healthcare education depends on its responsible and equitable use. Educators must not only harness AI to expand access, engagement, and efficiency but also guard against its potential to erode the humanistic and ethical foundations of rehabilitation practice (37). Preparing students for this evolving landscape requires a dual focus: cultivating technological proficiency and preserving the reflective, compassionate mindset that defines effective healthcare professionals.

## 5 Conclusion

AI presents healthcare education with both unprecedented opportunities and complex risks. It can enrich learning, support evidence appraisal, and prepare students for AI-enabled healthcare systems. However, without explicit ethical guardrails and structured competency frameworks, overreliance on AI threatens to erode critical thinking and professional judgment.

Healthcare educators must take the lead in shaping AI integration—ensuring it enhances rather than diminishes essential human skills. By embedding AI literacy, ethical practices, and cultural sensitivity into curricula, graduate programs can prepare students to navigate the future of healthcare with confidence, creativity, and integrity.

## Data Availability

The original contributions presented in the study are included in the article/Supplementary Material, further inquiries can be directed to the corresponding author.
